# Fast‐tracking dementia research: the comparison between Join Dementia Research, StepUp for Dementia Research and TrialMatch

**DOI:** 10.1002/alz70860_098466

**Published:** 2025-12-23

**Authors:** Yun‐Hee Jeon, Clare Shaw, Stephen Hall, Ed Park, Cheng‐Ya Lee, Mirim Shin, Martin N Rossor

**Affiliations:** ^1^ The University of Sydney, Camperdown, NSW, Australia; ^2^ NIHR Clinical Research Network, Leeds, United Kingdom; ^3^ Alzheimer's Association, Chicago, IL, USA; ^4^ NIHR Research Delivery Network, Leeds, United Kingdom; ^5^ UCL Institute of Neurology, London, United Kingdom

## Abstract

**Background:**

Recruiting participants in dementia research is often costly and time‐consuming, and its timely execution can be a significant determinant for a successful study. For the public who are interested in participating in dementia research, opportunities can be ad hoc. Delays in finding the right participants can result in studies taking longer to deliver, often requiring funding extensions, and ultimately increasing the cost and limiting the effectiveness of research and evaluation.

**Methods:**

Join Dementia Research (UK) and StepUp for Dementia Research (Australia) commenced their service to address these issues in 2014 and 2019 respectively. USA's TrialMatch was introduced in 2010 as an awareness tool where volunteers could come to the website and search for opportunities without giving any personal information. Unlike TrialMatch, JDR and StepUp use innovative matching technology and provide volunteers with an opt‐in, secure way of registering interest in dementia studies and allow researchers to access matched volunteers.

**Results:**

As of November 2024, JDR had >80,000 registrants (18.75% with dementia), supporting over 750 studies since its inception; while StepUp had >2100 registrants (7% with dementia), supporting >185 studies. Respective programme evaluation results suggest recruitment efficiency with a high level of satisfaction from users. However, both programmes have faced challenges with disparities in public participation in dementia research (e.g., gender, education and race/ethnicity). StepUp was modelled after JDR as an international collaboration, and yet their implementation and sustainability have met unique challenges in Australia (e.g., funding, culture, geographical characteristics). TrialMatch reported a substantial engagement with ∼2000 engagements a month through ∼800‐900 studies on the platform. Its ability to track the outcomes of their engagements is yet to be determined. Unique and common features of the programmes, key achievements, factors contributing to the success of recruiting volunteers to the programmes, as well as their role in fast tracking dementia research, will be discussed.

**Conclusion:**

The comparison of innovative technologies provides valuable insights for further enhancements of research participant recruitment platforms. A concerted and strategic effort is needed by leading registries to ensure narrowing volunteer participation gaps in dementia research, through for example the Dementia Research Recruitment Platform Global Collaborative (https://www.stepupfordementiaresearch.org.au/about‐2/).

